# High-Dose Deltamethrin Induces Developmental Toxicity in *Caenorhabditis elegans* via IRE-1

**DOI:** 10.3390/molecules28176303

**Published:** 2023-08-28

**Authors:** Chuhong Chen, Ying Deng, Linyan Liu, Zhenyan Zou, Chenzhong Jin, Zhiyin Chen, Shuanghui Wang

**Affiliations:** 1 Key Laboratory of Green Control of Crop Pests in Hunan Higher Education, Hunan University of Humanities Science and Technology, Loudi 417000, China; 2 Collaborative Innovation Center for Farmland Weeds Control Techniques and Application of Hunan Province, Hunan University of Humanities Science and Technology, Loudi 417000, Chinachenzhiyin100@163.com (Z.C.)

**Keywords:** deltamethrin, *Caenorhabditis elegans*, development, toxicity, endoplasmic reticulum unfolded protein response

## Abstract

Deltamethrin (DM), a Type II pyrethroid, is widely used worldwide in agriculture, household applications, and medicine. Recent studies have shown that DM exerts a variety of toxic effects on organs such as the kidney, heart muscle, and nerves in animals. However, little is known about the effects of high-dose DM on growth and development, and the mechanism of toxicity remains unclear. Using the *Caenorhabditis elegans* model, we found that high-dose DM caused a delay in nematode development. Our results showed that high-dose DM reduced the activation of the endoplasmic reticulum unfolded protein response (UPR^ER^). Further studies revealed that high-dose DM-induced developmental toxicity and reduced capacity for UPR^ER^ activation were associated with the IRE-1/XBP-1 pathway. Our results provide new evidence for the developmental toxicity of DM and new insights into the mechanism of DM toxicity.

## 1. Introduction

Deltamethrin (DM) is a Type II pyrethroid that is widely utilized as an insecticide and acaricide in various sectors, including agriculture, households, and medicine, on a global scale [1]. Initially, DM was believed to possess minimal toxicity towards mammals [2]. However, contemporary research has revealed that DM elicits numerous toxic effects on various organs, including the kidney, heart muscle, and nerves, in animals. Specifically, exposure to DM has been shown to induce apoptosis in goldfish kidney cells [3], promote inflammation and apoptosis in heart muscle cells, leading to heart damage in quail [4], reduce the number of neurons in mice [5], and cause immunotoxicity in silverfish upon acute exposure [6]. In summary, DM causes multiple toxic effects in animals.

The prevalence of DM exposure among humans is widespread, encompassing pregnant women and children [7]. Children, in particular, exhibit higher exposure levels to DM compared to adults, attributed to their increased contact with environmental surfaces and hand-to-mouth behaviors. Furthermore, children possess immature metabolic enzymes and blood-brain barriers, resulting in slower clearance of DM [8]. Epidemiological investigations have demonstrated that DM exposure negatively impacts neurodevelopment in children [9]. However, the effects of DM on growth and development remain poorly understood, and the mechanism of toxicity remains elusive.

Research on the cytotoxicity mechanism of DM has focused on redox-related signaling pathways, including the Nrf2/ARE pathway, caspase-dependent or -independent pathways, and the p66shc-mediated ROS production pathway [2]. However, several studies have shown that exposure to low-dose DM (<10 mg/kg) can induce endoplasmic reticulum (ER) stress in the hippocampus of mice, leading to impaired learning [5,10,11,12,13]. Additionally, acute exposure to DM (1 mg/L) has been shown to induce ER stress in *Carassius auratus*, resulting in decreased resistance to hypoxia [14]. Chronic exposure to DM was found to promote ER stress-induced apoptosis of neurons in quail brains [15]. Furthermore, DM (5 mg/L) has been shown to activate the AMPKα pathway through ER stress, leading to significant fat accumulation in mouse 3T3-L1 adipocytes and *Caenorhabditis elegans* [16]. In summary, low-dose DM administration leads to toxicity via the induction of ER stress. However, the effects of high-dose DM (>40 mg/L) on ER stress have not been reported, and it remains unclear whether DM induces developmental toxicity in juvenile animals via ER stress.

Globally, pesticides are used as a means of suicide, particularly in low- and middle-income countries, with pesticide suicides accounting for one fifth of suicides [17,18]. In addition, pesticide applicators and manufacturers are vulnerable to exposure to high doses of pesticides [19]. Therefore, it is of interest to study high-dose exposure to DM.

*C. elegans* is an excellent model organism for developmental studies due to its short lifespan, low maintenance requirements, 40–60% homology to humans, and extensive availability of mutant strains [16]. In this study, we used *C. elegans* as a model to investigate the effects of high-dose DM exposure during juvenile life on animal growth and development, as well as ER stress. Our findings provide new evidence for the developmental toxicity of DM and new perspectives on the mechanisms underlying DM toxicity.

## 2. Results

### 2.1. High-Dose DM Induces Developmental Delay in C. elegans

**Figure 1:**
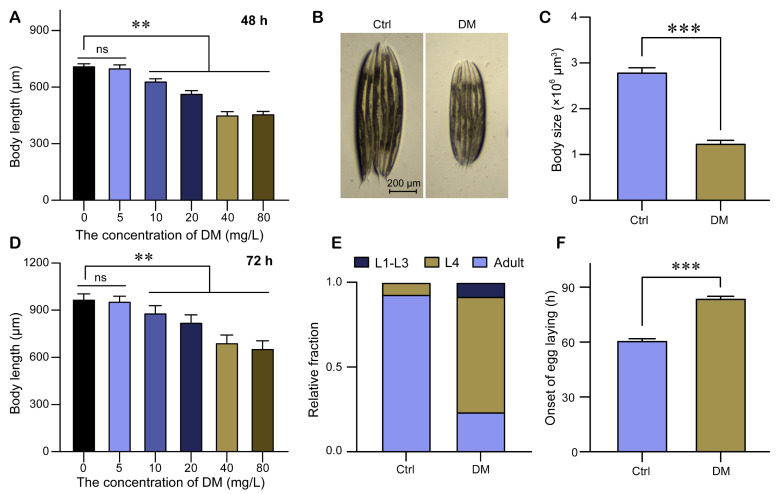
High-dose deltamethrin (DM) induces developmental delay in *Caenorhabditis elegans*. (**A**) Body length of *C. elegans* after exposure to different concentrations of DM from L1 for 48 h. (**B**) Representative picture showing the body size of *C. elegans* exposed to 40 mg/L DM from L1 for 72 h. (**C**) Body size of *C. elegans* exposed to 40 mg/L DM from L1 for 72 h. (**D**) Body length of *C. elegans* after exposure to different concentrations of DM from L1 for 72 h. (**E**) Quantification of different developmental stages of *C. elegans* exposed to 40 mg/L DM from L1 for 60 h. (**F**) Effect of DM exposure on the onset of egg laying. All error bars represent standard error of mean (SEM), and differences were considered significant at ** *p* < 0.01, and *** *p* < 0.001; ns, no significance.

To investigate the potential effects of DM on the developmental process, we conducted an experiment in which L1 larvae were exposed to various concentrations of DM and the length of the nematodes was subsequently assessed. Our results indicated that exposure to 5 mg/L DM for a period of 48 h had no discernible effect on the body length of *C. elegans*. However, exposure to DM concentrations ranging from 10–80 mg/L resulted in a significant reduction in nematode body length, with a dose-dependent relationship observed (Figure 1A). The trends observed after 72 h of exposure were largely consistent with those observed after 48 h (Figure 1D). Since the effects of 40 mg/L DM and 80 mg/L DM on *C. elegans* body length were nearly indistinguishable, we chose to use 40 mg/L DM as the treatment concentration for high-dose DM in our subsequent investigation. Because high-dose DM also affected nematode body width, we used body size as a measure of nematode size. The body size of high-dose DM (40 mg/L) was less than half that of the control (Figure 1B,C). To further investigate the effect of DM on nematode development, we examined the effect of DM on the proportion of larvae at each stage. In the DM-free environment, the majority of nematodes matured to adults within 60 h, with less than 10% remaining in the L4 stage. However, exposure to high-dose DM resulted in approximately 70% of nematodes remaining in the L4 stage after 60 h (Figure 1E). The onset of egg-laying time serves as an indicator of nematode reproductive development, and our results showed that high-dose DM significantly prolonged this period (Figure 1F). In conclusion, our results indicated that high-dose DM induced a developmental delay in *C. elegans*.

### 2.2. Exposure to High-Dose DM during Development Adversely Affects Healthy Physical Indicators in C. elegans

**Figure 2:**
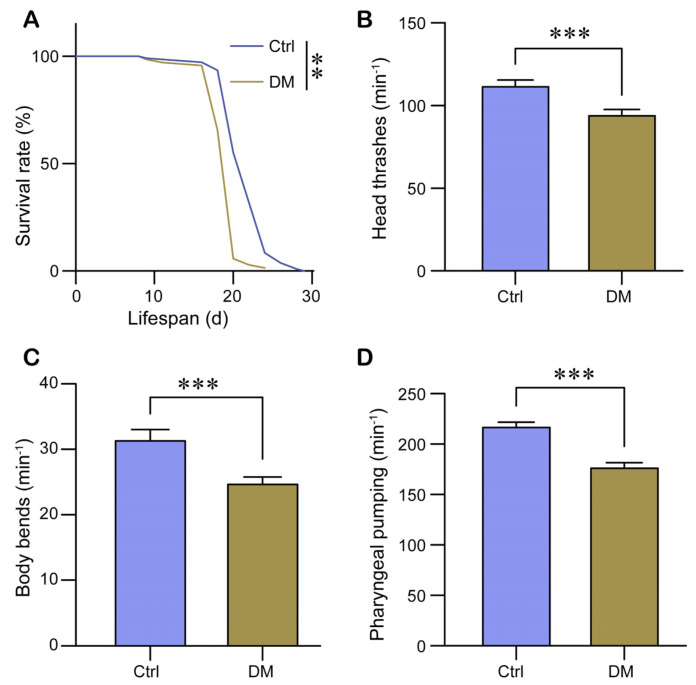
Exposure to high-dose DM during development adversely affects healthy physical indicators in *C. elegans*. The survival curve (**A**), head thrashes (**B**), body bends (**C**), and pharyngeal pumping (**D**) were assessed following exposure to 40 mg/L DM from L1 for a duration of 72 h. All error bars represent SEM, and differences were considered significant at ** *p* < 0.01, and *** *p* < 0.001.

The influence of early developmental stages on individual aging is significant [20]. To investigate the potential influence of DM exposure during development on nematode aging, we tested the lifespan, head thrashing, body bending, and pharyngeal pumping rate of nematodes exposed to DM for three days. Our results indicate that exposure to DM during the larval stage alone is sufficient to reduce the lifespan of nematodes (Figure 2A) as well as significantly reduce the frequency of head thrashing and body bending (Figure 2B,C). In addition, DM exposure also resulted in a slowing of the pharyngeal pumping rate of nematodes (Figure 2D). In conclusion, exposure to DM during development negatively affects nematode health indicators.

### 2.3. High-Dose DM Reduces the Ability to Activate the Endoplasmic Reticulum Unfolded Protein Response (UPR^ER^)

**Figure 3:**
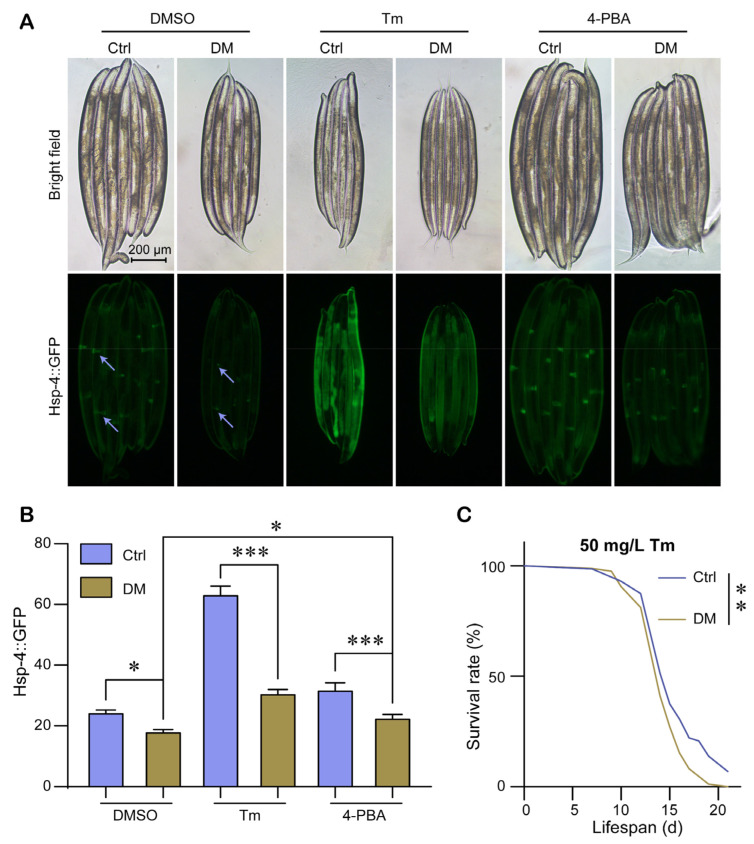
High-dose DM reduces the ability to activate the endoplasmic reticulum unfolded protein response (UPR^ER^). (**A**) Fluorescence images showing the *hsp-4p::GFP* expression level of *C. elegans* exposed to 40 mg/L DM from L1 for 72 h. The blue arrow points to the fluorescent highlight on the nematode’s abdomen. (**B**) Relative fluorescence density of *hsp-4p::GFP* worms exposed to 40 mg/L DM and supplemented with dimethyl sulfoxide (DMSO), 10 mg/L tunicamycin ©, or 5 mM 4-phenylbutyric acid (4-PBA). (**C**) Adult worms treated with 40 mg/L DM from L1 for 3 d were transferred to plates containing 50 mg/L Tm and survival was monitored. The blue arrow points to the fluorescent highlight on the nematode’s abdomen. All error bars represent SEM, and differences were considered significant at * *p* < 0.05, ** *p* < 0.01, and *** *p* < 0.001.

Previous studies have shown that low-dose DM induces ER stress and activates UPR^ER^ [21]. To test whether high-dose DM induces ER stress, we used the fluorescent strain SJ4005 (*hsp-4p::GFP*) with the ER stress reporter gene *hsp-4p::GFP* to determine the effect of DM on ER stress. Interestingly, high-dose DM did not increase the expression of *hsp-4p::GFP* but instead decreased the expression of *hsp-4p::GFP*; in particular, the brightness of two fluorescent spots on the abdomen of the nematode was significantly reduced (Figure 3A,B). We suspected that high-dose DM may have reduced the ability of nematodes to activate UPR^ER^, and we treated nematodes with 10 mg/mL tunicamycin (Tm), an inducer of ER stress, to test the ability of nematodes to activate UPR^ER^. We found that *hsp-4p::GFP* expression increased approximately 2.6-fold after supplementation with Tm when not exposed to DM, whereas *hsp-4p::GFP* expression increased only approximately 1.6-fold after supplementation with Tm when exposed to high-dose DM (Figure 3A,B). This suggests that DM reduces the ability of the nematode to activate the UPR^ER^. We also found that when exposed to 5 mM 4-phenylbutyric acid (4-PBA, a consistent inhibitor of the ER stress), DM still reduced the expression of *hsp-4p::GFP*, but the expression of *hsp-4p::GFP* was slightly higher in the DM+4-PBA group than in the DM group (Figure 3A,B). Dillin et al. [22] showed that the reduced activation capacity of the UPR^ER^ leads to reduced resistance of nematodes to ER stress. We found that nematode survival in 50 mg/L Tm was significantly reduced after DM treatment (Figure 3C). In conclusion, our results suggest that high-dose DM treatment reduces the activation capacity of nematode UPR^ER^.

### 2.4. High-Dose DM Reduces Transcript Levels of UPR^ER^ Target Genes

**Figure 4:**
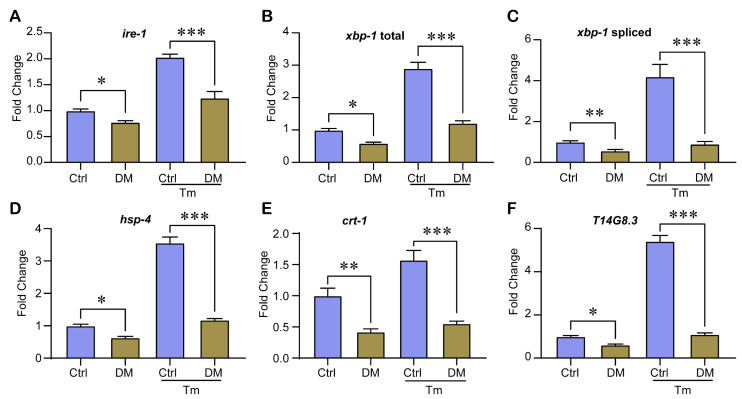
High-dose DM reduces transcript levels of UPR^ER^ target genes. Transcript levels of *ire-1* (**A**), *xbp-1* total (**B**), *xbp-1* spliced (**C**), *hsp-4* (**D**), *crt-1* (**E**), and *T14G8.3* (**F**) in worms treated with 40 mg/L DM from L1 for 3 d were measured by quantitative RT-PCR. All error bars represent SEM, and differences were considered significant at * *p* < 0.05, ** *p* < 0.01, and *** *p* < 0.001.

To progressively verify the effect of DM treatment on UPR^ER^ activation capacity, we measured the transcript levels of UPR^ER^ regulatory and target genes by quantitative RT-PCR. DM treatment decreased the basal transcript levels of the UPR^ER^ target genes *ire-1, xbp-1* total, *xbp-1* spliced, *hsp-4*, *crt-1*, and *T14G8.3* (Figure 4A–D). After TM treatment, the control *ire-1*, *xbp-1* total, *xbp-1* spliced, *hsp-4*, *crt-1*, and *T14G8.3* transcript levels increased by approximately 100%, 190%, 320%, 250%, 60%, and 440%, respectively, while the treated group increased by only approximately 60%, 100%,60%, 85%, 40%, and 80%, respectively (Figure 4A–D). This indicates that under ER stress, high-dose DM treatment reduces the transcript levels of UPR^ER^ target genes.

### 2.5. Developmental Toxicity of High-Dose DM in C. elegans Associated with ER Stress

**Figure 5:**
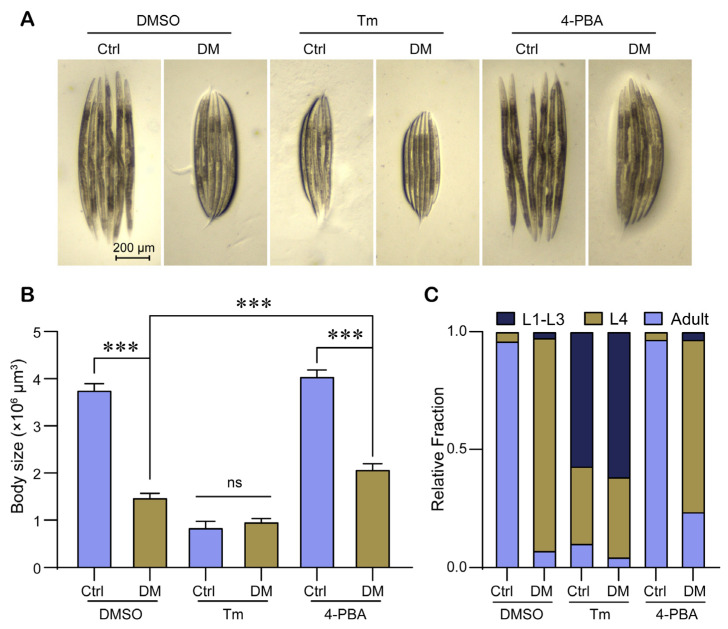
Developmental toxicity of high-dose DM in *C. elegans* associated with ER stress. (**A**) Representative images showing the body size of *C. elegans* exposed to 40 mg/L DM supplemented with DMSO, 10 mg/L Tm, or 5 mM 4-PBA from L1 for 72 h. (**B**) Body size of *C. elegans* exposed to 40 mg/L DM and supplemented with DMSO, 10 mg/L Tm, or 5 mM 4-PBA from L1 for 72 h. (**C**) Quantification of different developmental stages of *C. elegans* exposed to 40 mg/L DM and supplemented with DMSO, 10 mg/L Tm, or 5 mM 4-PBA from L1. All error bars represent SEM, and differences were considered significant at *** *p* < 0.001; ns, no significance.

Previous results in this study have shown that high-dose DM induces developmental defects in nematodes while reducing the expression level of the ER stress marker protein HSP-4 (Figure 1 and Figure 2). To confirm whether DM-induced developmental toxicity is related to ER stress, we tested the effect of DM on nematode body size development in the presence of Tm or 4-PBA. We found that DM could not further reduce nematode body size in the presence of 10 mg/L Tm; 5 mM 4-PBA could slightly restore the body size reduction induced by high-dose DM (Figure 5A,B). To further demonstrate that the developmental toxicity of high-dose DM on nematodes was related to ER stress, we tested the effect of DM on the distribution of larval stages. The proportion of each larval stage under Tm stress was not significantly different between DM treatment and the control. The 4-PBA treatment slightly increased the proportion of adults under DM treatment (Figure 5C). In conclusion, our results suggest that the developmental toxicity induced by high-dose DM is associated with ER stress.

### 2.6. High-Dose DM Induces Developmental Toxicity in C. elegans via IRE-1

**Figure 6:**
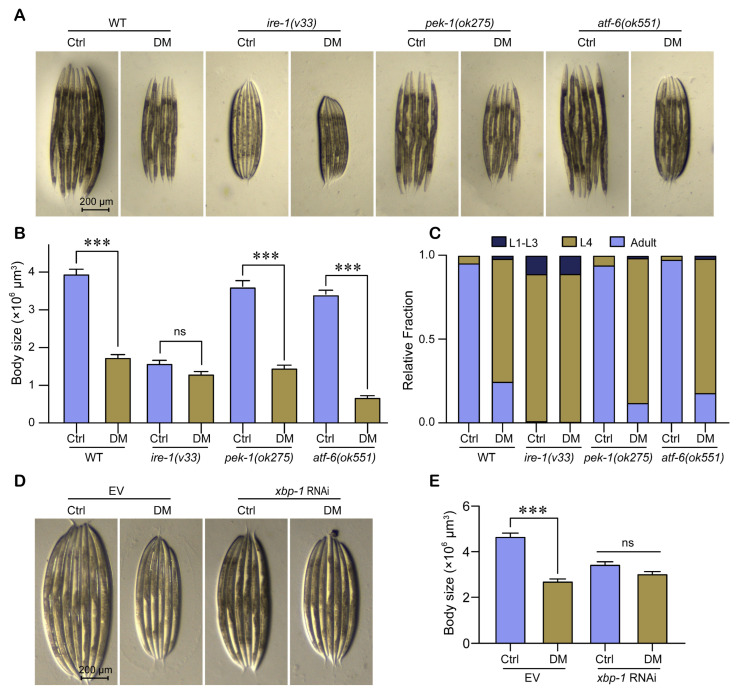
High-dose DM induces developmental toxicity in *C. elegans* via IRE-1. (**A**) Representative images showing the body size of wild type (WT), *ire-1 (v33)*, *pek-1 (ok275)*, and *atf-6 (ok551)* worms exposed to 40 mg/L DM from L1 for 72 h. (**B**) Body size of wild type (WT), *ire-1 (v33)*, *pek-1 (ok275)*, and *atf-6 (ok551)* worms exposed to 40 mg/L DM from L1 for 72 h. (**C**) Quantification of different developmental stages of WT, *ire-1 (v33)*, *pek-1 (ok275)*, and *atf-6 (ok551)* worms exposed to 40 mg/L DM from L1. (**D**) Representative images showing the body size of WT worms grown on empty vector (EV) and *xbp-1* RNAi exposed to 40 mg/L DM from L1. (**E**) Body size of WT worms grown on EV and *xbp-1* RNAi exposed to 40 mg/L DM from L1. All error bars represent SEM, and differences were considered significant at *** *p* < 0.001; ns, no significance.

Using the UPR^ER^ branching transmembrane regulator mutant strains RE666 (*ire-1(v33)*), RB545 (*pek-1(ok275)*), and RB772 (*atf-6(ok551)*), we determined the effect of high-dose DM on the development of each mutant strain. Our data showed that the effect of DM on the body size of *pek-1(ok275)* and *atf-6(ok551)* mutants remained significantly reduced in nematode body size as in wild type (WT); interestingly, DM did not affect the body size of the *ire-1(v33)* mutant (Figure 6A,B), suggesting that the reduction in nematode body size by high-dose DM was related to IRE-1 but not to PEK-1 and ATF-6 independently. In addition, DM significantly reduced the growth rate of WT, *pek-1(ok275)*, and *atf-6(ok551)* mutants, and after 60 h of development, the vast majority of the control group developed into adults, while most of the DM-treated group stagnated at the L4 larval stage; however, DM did not affect the growth rate of *ire-1(v33)* mutants, and after 60 h of development, approximately 80% of both the control and DM-treated groups remained at the L4 larval stage (Figure 6C). These results suggested that the developmental toxicity of high-dose DM on nematodes was related to IRE-1.

Activation of the IRE1 pathway leads to sequential splicing of the transcription factor XBP1 via IRE1 endonuclease activity. The spliced XBP1 is translated and activates a range of transcriptional targets essential for ER protein homeostasis [23]. To further validate that the developmental toxicity of high-dose DM is related to IRE-1, we performed RNA interference (RNAi) of *xbp-1* to reduce the level of XBP-1 spliced. We found that RNAi of *xbp-1* reduced the effects of high-dose DM on body size development. In conclusion, our results suggest that the developmental toxicity of high-dose DM is associated with the IRE-1/XBP-1 signaling pathway.

### 2.7. The Ability of High-Dose DM to Reduce UPR^ER^ Activation Is Associated with IRE-1/XBP-1

**Figure 7:**
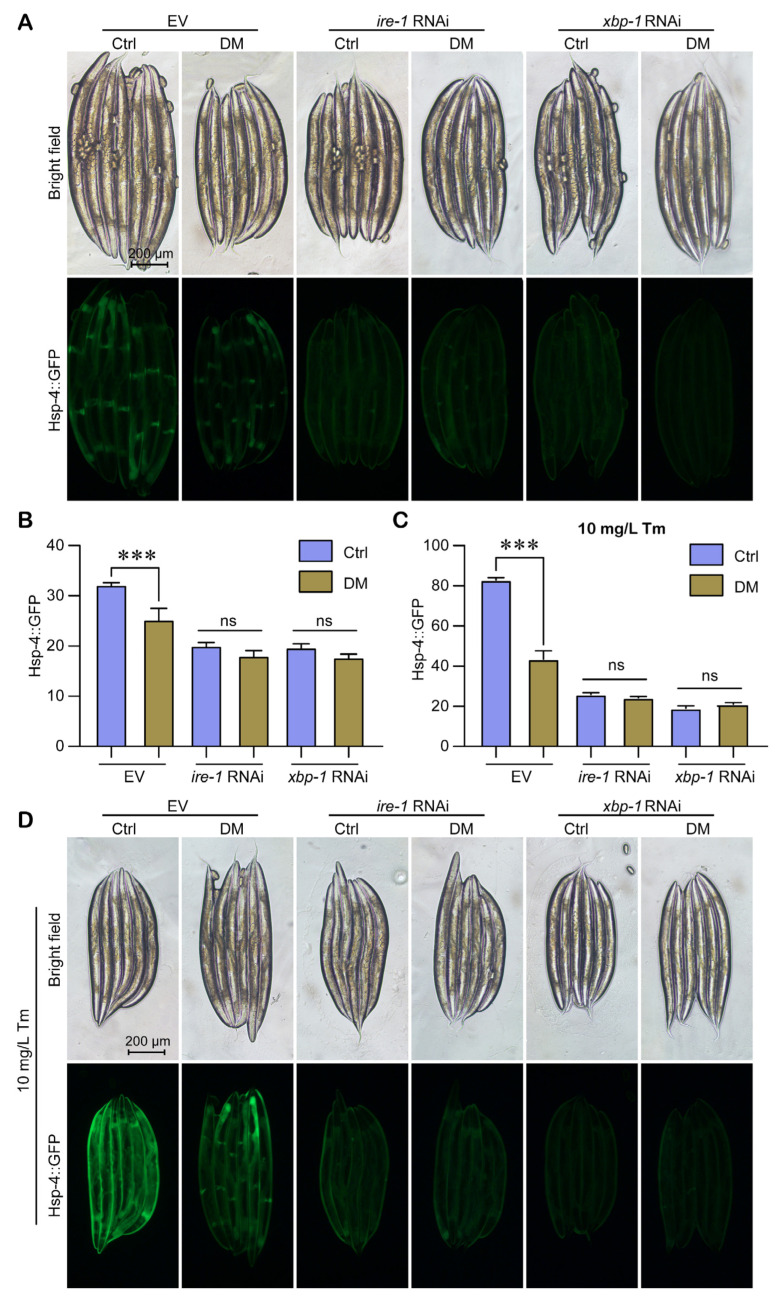
The ability of high-dose DM to reduce UPR^ER^ activation is associated with IRE-1/XBP-1. (**A**) Fluorescence images showing the *hsp-4p::GFP* expression level of *C. elegans* grown on EV, *ire-1* RNAi, and *xbp-1* RNAi exposed to 40 mg/L DM from L1 for 72 h. (**B**) Relative fluorescence density of *hsp-4p::GFP* worms grown on EV, *ire-1* RNAi, and *xbp-1* RNAi exposed to 40 mg/L DM from L1 for 72 h. (**C**) Relative fluorescence density of *hsp-4p::GFP* worms grown on EV, *ire-1* RNAi, and *xbp-1* RNAi exposed to 40 mg/L DM from L1 supplemented with 10 mg/L Tm for 72 h. (**D**) Fluorescence images showing the *hsp-4p::GFP* expression level of *C. elegans* grown on EV, *ire-1* RNAi, and *xbp-1* RNAi exposed to 40 mg/L DM from L1 supplemented with 10 mg/L Tm for 72 h. All error bars represent SEM, and differences were considered significant at *** *p* < 0.001; ns, no significance.

Meanwhile, we used RNAi to reduce the levels of IRE-1 and spliced XBP-1. In the EV group, high-dose DM significantly inhibited UPR^ER^ activation, whereas in the *ire-1* RNAi and *xbp-1* RNAi groups, the inhibitory effect of high-dose DM on UPR^ER^ was absent (Figure 7A,B). After supplementation with Tm, we found similar results, and the inhibitory effect of high-dose DM on UPR^ER^ was even more significant (Figure 7C,D). These results suggest that the ability of high-dose DM to reduce the activation of UPR^ER^ is related to the IRE-1/XBP-1 pathway, which is consistent with the mechanism of developmental toxicity of high-dose DM.

## 3. Discussion

In this study, we found that high-dose DM caused a delay in the development of *C. elegans*. We also found that high-dose DM reduced UPR^ER^ activation in *C. elegans*. Mechanistically, high-dose DM-induced developmental toxicity and reduced capacity for UPR^ER^ activation were associated with the IRE-1/XBP-1 pathway in *C. elegans*.

DM is a widely used pesticide, but its environmental and biological effects are of great concern. Studies have shown that DM is potentially harmful to animal development. In insects, DM exposure during the larval stage reduced body weight and prolonged development in *Chrysomya megacephala* [24]. In fish, exposure of zebrafish embryos to low-dose DM for 96 h significantly reduced larval body length and swim bladder [25]; it also resulted in defective larval locomotion in zebrafish [26]. In mammals, DM exposure caused weight loss in mice [27] and inhibited neuronal cell proliferation, maturation, and differentiation in mouse fetuses [28]; epidemiological studies have shown that DM affects brain development in children [9]. Our studies in nematodes showed that low-dose DM (5 mg/L) did not affect nematode size development, but high-dose DM (40 mg/L) significantly reduced nematode size, slowed developmental rates, and prolonged time to first oviposition (Figure 1). We demonstrated the developmental toxicity of high-dose DM in *C. elegans* for the first time, providing new evidence for the developmental toxicity of DM.

Multicellular animals face constant stress during growth and development, and to maintain cellular homeostasis, cells have evolved several subcellular stress responses, such as the cytoplasmic heat shock response, the UPR^ER^, and the mitochondrial unfolded protein response [29]. The ER activates the UPR^ER^ in response to intra- and extracellular stresses to restore ER homeostasis [30]. Studies have shown that DM induces ER stress. In mammals, DM (1–2.5 mg/kg) caused ER stress in mouse hippocampal neurons with elevated levels of C/EBP homologous protein (CHOP) and glucose-regulated protein 78 (GRP78) [12]; oral administration of DM (3 mg/kg) to mice resulted in ER stress-mediated neuroinflammation [13]; and DM (5 mg/kg) exposure caused ER stress and learning deficits in the hippocampus of adult mice [10]. Oral administration of DM (1 or 3 mg/kg) to mice induced ER stress and activation of apoptotic signaling [5]. In birds, DM (30 mg/kg) exposure significantly increased the expression of ER stress-related mRNA in quail brain tissue [15]. In fish, DM exposure (0.61–4.88 μg/L) caused gill damage in crucian carp through a mechanism related to activation of ER stress [14]. In cells and *C. elegans*, DM (5 mg/L) significantly increased fat accumulation through the ER stress-AMPKα pathway [16]. In conclusion, previous studies have shown that low-dose DM (<40 mg/L) induces ER stress, resulting in toxicity.

Interestingly, our study showed that high-dose DM (40 mg/L) decreased the expression level of ER stress marker protein Hsp-4 (Figure 3A,B). We suspected that this might be due to the fact that DM reduced the activation capacity of the UPR^ER^. Therefore, we used Tm to induce ER stress and tested the effect of DM on UPR^ER^ under Tm exposure. The results showed that DM reduced the level of UPR^ER^ under ER stress (Figure 3A,B). Taylor and Dillin [22] showed that the reduced activation capacity of UPR^ER^ leads to a decreased resistance to ER stress. Our results also suggest that DM reduces the ability to survive under Tm exposure (Figure 3C). Furthermore, we measured the transcript levels of UPR^ER^ regulatory and target genes and found that DM reduced the transcript levels of UPR^ER^ regulatory genes and target genes *ire-1, xbp-1* total, *xbp-1* spliced, *hsp-4, crt-1*, and *T14G8.3* under ER stress (Figure 4). Finally, we found that *ire-1* and *xbp-1* RNAi rescued the reduction of UPR^ER^ activation capacity by DM (Figure 7). Thus, our results fully demonstrate that high-dose DM does not induce ER stress but reduces the activation capacity of UPR^ER^. Our results provide a theoretical basis for the development of drugs for high-dose DM exposure, in particular UPR^ER^-targeted therapy for DM pesticide suicide. Administration of drugs that enhance UPR^ER^ activation may be a viable option to mitigate high-dose DM toxicity.

The ability to activate the UPR^ER^ decreases with age [22]. Abnormal accumulation of misfolded or unfolded proteins is a hallmark of senescence, and a decrease in the ability to activate UPR^ER^ leads to abnormal accumulation of unfolded proteins [31]. Therefore, the decrease in UPR^ER^ activation capacity by DM treatment may increase the abnormal accumulation of misfolded or unfolded proteins and accelerate nematode senescence. Our finding that DM exposure during larval stages shortened lifespan and negatively affected healthy physical indicators such as head thrashing, body bending, and pharyngeal pumping rate provides evidence that DM accelerates senescence (Figure 2).

UPR^ER^ is composed of three conserved branches that transmit ER stress signals to the cytoplasm through the transmembrane regulators inositol-requiring enzyme 1 (IRE1, homologous to nematode IRE-1), PKR-like ER kinase (PERK, homologous to nematode PEK-1), and activating transcription factor 6 (ATF6, homologous to nematode ATF-6) to activate different UPR^ER^ signaling pathways [32]. One study showed that gill damage in *Carassius auratus* due to DM exposure was associated with PERK and IRE1 [14]. We found that *pek-1* and *atf-6* mutations did not alter the effects of high-dose DM on body size development and developmental rate in *C. elegans*, while high-dose DM was not toxic to *ire-1* mutants and *xbp-1* RNAi nematodes (Figure 6). This suggests that high-dose DM causes developmental retardation in nematodes via IRE-1. However, our findings are limited to *C. elegans*, and further validation of our conclusions in mice and epidemiology is needed. In addition, our study did not investigate why high-dose DM reduced UPR^ER^.

## 4. Materials and Methods

### 4.1. Nematode Strains and Maintenance

All strains were grown at 20 °C using standard nematode methods as described previously [33]. Unless otherwise stated, standard nematode growth medium (NGM) inoculated with *E. coli* OP50 was used for normal growth. N2, RE666 (*ire-1(v33)*), RB545 (*pek-1(ok275)*), SJ4005 (*hsp-4p::GFP*), and RB772 (*atf-6(ok551)*) strains were obtained from the *Caenorhabditis elegans* Genetics Center (CGC; University of Minnesota, Minneapolis, MN, USA). The synchronization method for *C. elegans* was the bleaching method [34].

### 4.2. Drugs and Treatment

Deltamethrin (DM), tunicamycin (Tm), and 4-phenylbutyric acid (4-PBA) were purchased from Shanghai Aladdin Biochemical Technology Co. (Shanghai, China). DM was dissolved in distilled water, and Tm and 4-PBA were dissolved in dimethyl sulfoxide (DMSO), which was used as a control. Except for the DM screening experiment, DM was used at a concentration of 40 mg/L for all other experiments, 10 mg/L for Tm in the larval stage, 50 mg/L in the adult stage, and 5 mM for 4-PBA. DM, Tm, 4-PBA, and other drugs were added to OP50 food and then applied to NGM solid plates. Unless otherwise noted, all drugs were applied from synchronized larval L1. 

### 4.3. Body Size Estimation of Nematodes

DM solution was added proportionally to OP50 food configured to contain 0 mg/L, 5 mg/L, 10 mg/L, 20 mg/L, 40 mg/L, and 80 mg/L DM and coated onto NGM plates. Synchronized L1 larvae were transferred to the above plates and incubated at 20 °C for 48 h or 72 h. After incubation, the nematodes were imaged using a stereomicroscope, and the body length (from the mouthparts to the end of the hindgut) and body width (the horizontal width of the gonad at the location of the gonad) of the nematodes were measured using ImageJ V1.8.0.112 software. Approximately 10 nematodes were measured for each treatment. A minimum of three biological replicates were performed for each group, and body size was estimated using Equation (1):

(1) $$\mathrm{Body}\mathrm{size}=\pi \times {\left(\frac{\mathrm{Body}\mathrm{width}}{2}\right)}^{2}\times \mathrm{Body}\mathrm{length}$$

### 4.4. Nematode Development Analysis

Synchronized L1 larvae were transferred to NGM petri dishes containing 0 mg/L or 40 mg/L DM for 60 h, after which the nematodes were quantified for the different developmental stages (L1–L3, L4, adult). At least three biological replicates were performed for each group.

### 4.5. Onset of Egg Laying

Synchronized L1 larvae were transferred to NGM petri dishes containing 0 mg/L or 40 mg/L DM and incubated for 48 h. Approximately 10 nematodes were picked and transferred to petri dishes containing the corresponding drug, placing 1 nematode in each dish 1, and the egg laying status was observed every hour. At least three biological replicates were performed for each group.

### 4.6. Lifespan Analysis

Nematode lifespan was determined using standard methods [35]. Synchronized L1 larvae were cultured in NGM dishes containing 0 mg/L or 40 mg/L DM. After 3 d, approximately 30 nematodes were plucked and transferred to dishes containing 5′-fluorodeoxyuridine (50 μM) without drugs for further incubation. The number of dead nematodes was counted every 2 d, and mortality was calculated until all nematodes were dead. Death was judged by the lack of response to gentle contact of the worm with a pick. At least 3 biological replicates were performed for each group.

### 4.7. Head Thrashing and Body Bending Analysis

Head thrashing and body bending frequency analysis was performed 3 days after drug treatment of L1 larvae. Approximately 10 nematodes were selected from each dish. Head thrashing and body bending frequency were determined using standard methods [36]. At least three biological replicates were performed for each group.

### 4.8. Pharyngeal Pumping Assay

Pharyngeal pump rate was measured after 3 d of drug treatment. The number of nematode pharyngeal pump contractions was observed for 60 s. Approximately 10 nematodes per treatment were randomly measured, and the experiment was repeated three times.

### 4.9. Measurement of UPR^ER^ Activation Levels

The activation level of UPR^ER^ was determined using the *Hsp-4p::GFP* strain. The nematodes were placed on gel pads containing 2% agarose after 3 days of 0 mg/L or 40 mg/L DM treatment, and 5 uL of 0.4 mM levamisole solution was added to paralyze them. Fluorescence images were captured using a Nikon ECLIPSE Ti-U fluorescence microscope (Nikon Group, Tokyo, Japan). The average fluorescence intensity was scored using Image J (ImageJ V1.8.0.112) software.

### 4.10. ER Stress Survival Analysis

Synchronized L1 larvae were transferred to Petri dishes containing 0 mg/L or 40 mg/L DM for 3 d. Approximately 30 nematodes per dish were selected and transferred to Petri dishes containing 50 mg/L Tm without DM for further incubation, and survival was assessed daily. The experiment was repeated at least three times.

### 4.11. Quantitative Real-Time PCR

Total RNA was extracted and purified from at least 1000 nematodes using the Total RNA Extraction Kit (Beijing Solarbio Science & Technology Co., Ltd., Beijing, China). cDNA was synthesized using the Universal RT-PCR Kit (M-MLV) (Beijing Solarbio Science & Technology Co., Ltd., Beijing, China). PCR was performed using the indicated primers as follows (Table 1). Act-1 was used as an endogenous control. The experiment was repeated at least three times.

### 4.12. RNA Interference (RNAi) in C. elegans

Double-stranded DNA containing homologs of the target gene was ligated to plasmid L4440 and transformed into a *E. coli* HT115 (DE3) receptor state. *E. coli* HT115 containing the appropriate vector was incubated in LB medium containing ampicillin (100 ug/L) on a shaker at 37 °C for 12 h, then IPTG (1 mM) was added, and the incubation continued for 3 h. The bacterial solution was coated on NGM dishes containing ampicillin and IPTG, and synchronized nematodes were transferred to the dishes for RNA interference. The bacterial solution was spread on NGM plates containing ampicillin and IPTG.

### 4.13. Statistical Analysis

The present study used independent experiments repeated at least three times. For data sets greater than two, statistical analysis was performed using one-way analysis of variance (ANOVA) followed by Tukey’s post-hoc test. Survival analysis was performed using the Kaplan-Meier test, and *p* values were obtained using the log-rank test. Data analysis and graphing were performed using GraphPad Prism 8 software. Error bars are represented by standard error of mean (SEM), and statistical significance was determined at * *p* < 0.5; ** *p* < 0.01; and *** *p* < 0.001; ns, no significance.

## 5. Conclusions

In conclusion, high-dose DM reduces the activation capacity of the UPR^ER^ and induces developmental retardation in *C. elegans* via IRE-1/XBP-1. Our results provide new evidence for the developmental toxicity of DM and new insights into the mechanisms of DM toxicity.

## Figures and Tables

**Table 1:** Primers used in real-time PCR.

Target Gene	Forward Primer	Reverse Primer
*ire-1*	TCCTCAACCGCTCCATCAACAT	TCCTCAACCGCTCCATCAACAT
*hsp-4*	GAACAACCTACTCGTGCGTTGG	GAACAACCTACTCGTGCGTTGG
*xbp-1* total	GGACTTCTTCGGCTTCTGGAGT	GGACTTCTTCGGCTTCTGGAGT
*xbp-1* spliced	GGTGGATGGAGGGAGAAGATT	GGTGGATGGAGGGAGAAGATT
*crt-1*	GAAGTAATAGCCGAGGGAAGC	GAAGTAATAGCCGAGGGAAGC
*T14G8.3*	CACCTCCATCAACAACAACAT	CACCTCCATCAACAACAACAT
*act-1*	GTCATGGTCGGTATGGGACA	AGTGAGGAGGACTGGGTGCT

## Data Availability

Data is contained within the article.

## Sample Availability

Samples of the compounds are not available from the authors.
